# Pathology, Practical Medicine, and Therapeutics

**Published:** 1837-10

**Authors:** 


### PATHOLOGY, PRACTICAL MEDICINE, AND THERAPEUTICS.

*An Account of Hernia Pericardii.* By T. Hart, a.b. &c., Dublin.

This is a short account of a curious pathological condition of the pericardium hitherto, we believe with Mr. Hart, unnoticed. It presented itself in an aged female subject with general anasarca, brought into the Park-street School for dissection.

"On opening the thorax, with a view to demonstrate the organs situated in this region, it was found that the pleural cavity on either side was so completely obliterated by old and extensive adhesions, as to render those parts totally unavailable for anatomical purposes. The anterior mediastinum was occupied by a membranous pyriform sac of considerable size, lying on and overlapping the pericardium, which was greatly distended, as well from the large quantity of fluid it contained, as from the hypertrophied condition of the heart, this organ being in a state of active aneurism. The sac contained fluid to the amount of three or four ounces, and was free and unattached in its whole extent, except at its upper and smaller extremity, where there existed a free communication between it and the pericardium, as proved by the following simple experiment:—By raising the sac off the pericardium, it was made to empty itself, the fluid passing into, and increasing the already distended condition of that sac; and again, on pressing on the pericardium from below upwards, the abnormal sac, in its turn, assumed its former dilated appearance. The pericardium was next laid open by a longitudinal incision, and its communication with the abnormal sac brought into view: it was situated immediately at the point of reflection of the pericardium on the aorta, in one of those pouches, designated the sinuses of Haller, and presented a regular, well defined circular orifice, freely admitting the introduction of a finger, around which the fibrous membrane of the pericardium formed a thick annulus of great strength, and then passed, considerably attenuated however, over the whole surface of the sac: the serous membrane passed into, and lined it throughout. The preparation of this very interesting case is now preserved in the Park-street Museum."

*Dublin Journal, July,* 1837.

### *On the Employment of Tartar Emetic in large Doses in Inflammation of the Mamma.* By J. C. W. LEVER, Esq.

SOME time since Dr. Evory Kennedy, of Dublin, recommended this practice, and in the present communication Mr. Lever relates six cases by himself, and two by Dr. Ashwell, in which it was employed with the greatest success. The general mode of administering the tartar emetic was first to give a cathartic draught containing one grain of this salt, and then giving about half a grain in a mixture every two hours. Mr. Lever is of opinion that "the patient must be *nauseated;* and this kept up for a longer or shorter time according to the state of the indurated or inflamed glands." [In using this remedy in *pneumonia,* its effect is certainly as beneficial, if not more so, when it does not nauseate. Inflammation of the mamma is so very obstinate and painful a disease, that we are pleased to receive any accession to the ordinary modes of treating it, and shall be glad to put in practice the treatment recommended by Mr. Lever.] *Med. Gazette. Aug.* 19.

---

### *On the Efficacy of the Cold Affusion in the Treatment of Poisoning with Sulphuric Acid.* By J. T. BANKS, M.D., Physician to the Louth Dispensary.

A GIRL, æt. nineteen, swallowed by mistake rather more than forty drops of hydrocyanic acid, which produced immediate convulsions and insensibility. After the convulsions, and while the patient was in a state of insensibility, "the eyes fixed and glistening, the pupils dilated and wholly insensible, the respiration slow and feeble, the pulse scarcely perceptible, with a cold clammy sweat over the surface," various stimulants, both external and internal, were employed without effect. At this time Dr. Banks directed "a stream of cold water from a large pitcher, at some distance above the head," to be poured upon it. "A minute had not elapsed before the patient began to move, and in a very short period longer she became convulsed, and writhed and moaned as if in agony." A repetition of the effusion had a similar effect, and the patient gradually rallied.—We join in the candid doubts of Dr. Banks as to whether the case would have proved fatal if no remedial means had been adopted, but think, with him, the case valuable, as proving the great power of the cold affusion "in arousing the nervous system when its energy seems almost destroyed." *Edin. Journal. July,* 1837.

---

### *On Violent Pulsations of the Aorta in the Epigastric Region, and their Treatment.* By W. FAUSSETT, A.B. Dublin.

THIS paper refers to a well-known disease, or rather symptom of disease, which is often very distressing to the patient and refractory to treatment. The author does not profess to add much to what is already known of the nature and causes of it, but his communication assumes a character of some importance, from containing what he states to be "a method of treatment which has been found eminently successful in the many cases in which he has tried it." We believe Mr. Faussett is correct in attributing many cases of this sort to that fruitful source of chronic malady, abdominal plethora.

The following summary, which concludes Mr. F.'s paper, contains his views of the nature of the affection:—

"1st. That violent pulsations of the aorta in the epigastrium, constitute an affection *not 'of little importance,'** and one that is attended with actual, not imaginary suffering. 2d. That this affection is not a *nervous* one in the received sense of the word, at least does not indicate a state of general nervous debility. 3d. That it is not an effect of which dyspepsia is the cause, but that these two, with many others, are conjoint effects, or links in a chain of morbid sympathies, all dependent on some common cause. 4th. That the several causes enumerated by authors, have led to confusion in estimating the true nature of the complaint, while

* So stated by Dr. Baillie.

the proximate cause, which is probably alike in all cases, has not been properly distinguished. 5th. That this proximate cause appears to be a deranged condition of the great nervous centre of the sympathetic, of its branches, or its anastamoses. 6th. That this hypothesis satisfactorily explains all the morbid phenomena of epigastric pulsations. 7th. That this hypothesis is corroborated: first, by the fact that some of the phenomena, *e. g.*, sense of faintness, sense of vital depression, extreme anxiety, prostration of strength, &c. are (in degree) like effects with what we know to follow from a direct lesion of the nervous centre, as evidenced by a blow upon the epigastrium; and secondly, by the fact that the carotid arteries form that portion of the vascular system, which, next to the aorta, is most subject to increased pulsations; at the same time that these vessels are, in some measure, circumstanced with respect to the large cervical ganglia, and their anastamoses with cerebral and spinal nerves, *as is* the aorta with respect to the plexuses in *its* vicinity. 8th. That the deranged condition of the great nervous centre of the sympathetic, its branches, and its anastamoses, is owing to a state of visceral congestion, or subacute inflammation; or is at least in some manner associated with an over-fulness of the vessels in this region. 9th. That those cases of epigastric pulsations which are caused by tumors pressing upon the course of the aorta, operate not merely by diminishing the vessel's caliber, but by irritating its coats; thus likewise producing morbid impressions upon the nervous tissues of the artery. 10th. That the state of the system in general is not what is commonly denominated '*below par*;' on the contrary, that there is rather a tendency to vascular fulness, which is proved, 1st, by a very frequent determination of blood to the head; 2d, by the vessels relieving themselves by capillary exhalation, as in the case of hydrocephalus; 3d, by the relief which general and local bloodletting, constantly afford. Lastly, that the affection is not only entirely within our control, but curable upon the simplest principles of medical science, always, however, premising, that the coats of the vessel have not undergone any particular organic change."

The following is the author's treatment.

"The plan of treatment is as follows: local bleeding by cupping and leeching, (a combination of these, leeching first and *then* cupping, answers the purpose well.) In a few cases general bloodletting is necessary, viz., those in which the symptoms are severe, and local abstraction of blood has been employed in vain, or where the pulse seems to warrant the practice; or without this criterion, where there is a sense of tightness, fulness, or uneasiness in the head,—in any modification of such cases, venesection from the arm to a moderate extent, may be practised with safety, the relief afforded the patient will be striking, and the blood will often be found buffed, and even cupped. After local bleeding, counter-irritation by means of antimonial ointment, or the croton oil, rubbed over the epigastrium, or between the shoulders, may be employed with benefit. The state of the digestive organs demands particular attention, if an obstruction be suspected in the arch of the colon from an accumulation of fæces there, or in any other part of the intestines, a tolerably active purgative had best be first exhibited and mild aperients afterwards. But as soon as local bleeding has been *first* practised, and the bowels moved, we should at once have recourse to the use of blue pill, combined with sedatives and James's powder, or *hippo* [Anglicè Ipecacuan.], and give it twice or thrice daily, until the mouth becomes slightly affected. The inspissated juice of cicuta, or henbane and hippo, are what I have conjoined with blue pill, and derived the greatest advantage from. If the urgency of the symptoms demand it, and it be thought desirable to expedite the effect of the mineral upon the mouth, mercurial inunction either to the inner part of the thighs, or over the abdomen, should be resorted to, for it will invariably be found, the moment the gums become tender, and seldom before this period, that all the symptoms are mitigated. A strict regimen must be also enjoined; meat, wine, and stimulants, must be forbidden, and contrary to what is imagined, and usually practised, the patient's diet should be made to consist of milk, and farinaceous vegetables; barley, arrow-root, sago, &c. In the course of a few days, (averaging at about a fortnight,) and often contemporaneously with the mouth becoming sore, it will be found that the epigastric pulsations have ceased, that the appetite and digestive functions have improved, the patient's spirits returned, and a general renovation taken place in his state of health.

*Dublin Journal. July*, 1837.

---

*Observations on the Character and Treatment of the Spotted Fever, at present existing in St. Giles's and the Neighbourhood.* By John Wilson, m.d. Physician to the Middlesex Hospital.

This paper contains the most decided and interesting illustration that we have yet met with of the good effect of Dr. Stevens's saline treatment in diseases attended with a morbid, or, as it is usually termed, dissolved state of the blood.

The following is an account of the general symptoms of this fever on the admission of the patients to the hospital:

"Countenance dusky; the worst had the livor of pneumonia; one more inclined to the purple of asthma or emphysema, without cough or expectoration. Spots more or less over all the body, especially the trunk, varying in degree and extent; some faint, like a dark marked case of measles; in others the spots were larger, more confluent, and of a darker hue, mottling the trunk and extremities in a great variety of forms and shades. Pulse generally below one hundred; stupor and drowsiness, but sensible on being roused for the most part. Urine diminished; a few had it turbid, mostly deep-coloured. No purging. Skin hot and dry; tongue dry, brown or red, often with sordes."

The following is the account of the examination of a body sixteen hours after death; and the appearances corresponded with those generally observed in fatal cases previously to the adoption of the saline treatment.

"The whole body still warm and covered with port-wine-like stains. The cavities, particularly the thorax, smoked when laid open; all the blood was quite fluid and black, staining the lining membrane of the heart and veins. The lungs were gorged with blood, and in some parts it was extravasated into the tissue in lumps, which sunk in water. A large quantity of clear fluid in both lateral ventricles of the brain. Mucous membrane of the intestines healthy, as well as all other parts of the body."

In consequence of the ill success attending his previous method of treating this fever, Dr. Wilson very properly resolved to change it; and the following extracts give an account of the new plan and its results.

"Finding that the blood in all the cases was fluid, and of a black venous colour, and knowing that we could change the dark venous to a bright arterial coloured blood, we determined, should we have any more similar cases, to give Dr. Stevens's saline treatment a fair trial, but at the same time not to abandon the use of the warm bath, which we are in the habit of giving to all fever, and every other case, on admission, excepting some head or heart affections."

From the 4th of May till the 16th of June, nineteen cases of the fever were admitted, from whom the description of the symptoms given above was taken.

"Now all the nineteen cases, to which these symptoms apply in a general way, were on admission put into a warm bath, and well washed with soap, had their heads shaved, and to which cold lotion was occasionally applied; afterwards all had Dr. Stevens's powder which he gave in cholera, viz. carbonate of soda, ʒss.; muriate of soda, ℈j.; chlorate of potass, gr. vj. This was repeated every six hours in a cupful of water, or more if they liked. The bath and powder applied to one and all. Some, when they became insensible or delirious, and refused the powders on account of their taste, had in their place, by way of common drink, a ʒj. of the chlorate of potass in a quart of water every twenty-four hours; and these salines were continued till convalescence." . . . . "The powders did not irritate the stomach, and acted sufficiently on the bowels, promoting, at the same time, the secretions of the skin and kidneys; and the spots on the body, from a livid, became of a brighter hue, some of a bright red. The urine for the first days was acid, clear, and dark coloured; in some afterwards it became alkaline, in others neutral; in all it increased in quantity, and became more pale, but always clear, and continued so during the convalescence; after the powders had been stopped, it again became acid. The bowels in almost all were regular." . . . . "Evidences of recovery were a bright eye and clear countenance; increase of urine; moist skin; bowels acting regularly every twenty-four hours; sordes disappearing. These changes were generally preceded by sleep of two or three days and nights, after which sleep recovery became secure. The port-wine stains and rubeoloid spots changed from a dusky to a brighter red colour, and then gradually, but slowly, disappeared."

Two of the nineteen cases treated with the saline medicines died, and it is remarkable that the blood found in these, on dissection, was of *a bright red colour*, instead of being black, as in those in which the saline treatment was not employed.

*Med. Gazette. July* 1, 1837.

---

*Report of St. John's Fever and Lock Hospitals, Limerick.*
By W. J. Geary, M.D.

This is a valuable paper, and affords some additional materials to the statistics of fever, the more worthy the attention of the medical enquirer from the length of time and the great number of cases comprehended in the Report. We have only room for the tabular summary with which the paper concludes.

| Year. | Admitted | Cured. | Died. | Average Mortality. |
|---|---|---|---|---|
| From 1794 to 1819 | 10,954 | 10,336 | 618 | 1 in 17½. |
| From 1820 to 1836 | 22,682 | 21,338 | 1,344 | 1 in 16. |
| Total ......... | 33,636 | 31,674 | 1,962 | 1 in 17. |

*Dublin Journal. July*, 1837.

---

*Cure of Gout by Colchicum applied externally.*
By T. W. Wansbrough, Esq., Fulham.

[The following facts deserve to be recorded, and the practice further tested: we fear future trials will prove the result in this case to have arisen from individual idiosyncrasy.]

A gentleman who had previously had two attacks of gout, met with an accident which dislocated and fractured the fibula.

"Thirty-six hours after the accident, gout appeared in the great toe of the wounded foot, and increased with such violence, that, from intense suffering, at the end of six hours, the patient became exhausted and slightly delirious. Every application that could be thought of was used without affording any relief; on the contrary, each appeared to increase the pain; even colchicum, taken internally, totally failed. As a dernier resort, I added ʒij. of tinct. *sem. colch.* to a ℥iv. spirit lotion last used. The effect was magical; immediate relief was felt, and, after two or three applications, the patient fell asleep, slept soundly for four hours, and awoke free from pain, nor has he had a return of gout since, save some compunctious twitchings, once or twice, which were immediately removed by a few drops of the tinct. colch. applied to the part, through the stocking."

An anonymous correspondent, in the next Number of the same Journal, states a similar result to attend the local use of morphia. "The treatment is this:—Bathe the part in hot water for one minute, and then apply lint, spread with simple cerate, on which is spread about three grains of the acetate of morphia. The change which speedily takes place is surprising. The patient, from extreme agony, at once passes to a state of comfort."

*Lancet. July* 29 and *Aug.* 5, 1837.

---

*On the Existence of a "Cerebral Murmur" in Chronic Affections of the Brain.*
By J. R. Smyth, M.D.

Dr. Smyth here details three cases of chronic hydrocephalus in which this phenomenon was detected, the patients being one year, one and a half, and seven years respectively. In the oldest child, the sutures on the upper part of the head were all un-united. The following is Dr. S.'s description of the sounds:—

"On applying the ear to any of these situations, and also over the parietal bones, a brief, rather soft, rushing sound, synchronous with the pulse, is distinctly audible. Over the anterior fontanel and parietal bones it is heard the loudest, and it gradually becomes fainter as the examination recedes over the sagittal suture, posterior fontanel, and occipital bone. To be somewhat more particular—in the earliest description of this new auscultic phenomenon, it is an abrupt, brief, rushing, arrested sound, in tone something between a *bruit de soufflet* and a *bruit de rape;* not soft enough for the former, nor hard enough for the latter. In its character of intensity it varies of course with the energy of the action of the heart and pulse. When the circulation is excited and vigorous, and the heart, unembarrassed by palpitation, beats steadily and strongly, the sound is most clearly audible."

[This sound, we presume, is a mere bruit de soufflet, having its seat in the arteries within the skull, analogous to that so frequently produced by the subclavian arteries. It may possibly be made available in the diagnosis of cerebral diseases; but we cannot at all join with Dr. Smyth in expecting that any "auscultatory signs will enable us to mark the progress of the effusion into the ventricles of the brain, with the same degree of accuracy as in hydrothorax and empyema." The circumstances of the two cases are in no respect analogous. This "cerebral murmur" was some years since noticed by an American physician.]

*Med. Gazette. Aug.* 19, 1837.

